# Supplementary figures and images for: A hierarchy of metabolite exchanges in metabolic models of microbial species and communities
Source: PLoS Comput Biol. 2024 Sep 26;20(9):e1012472. doi: 10.1371/journal.pcbi.1012472 (PMC11460683; doi:10.1371/journal.pcbi.1012472)

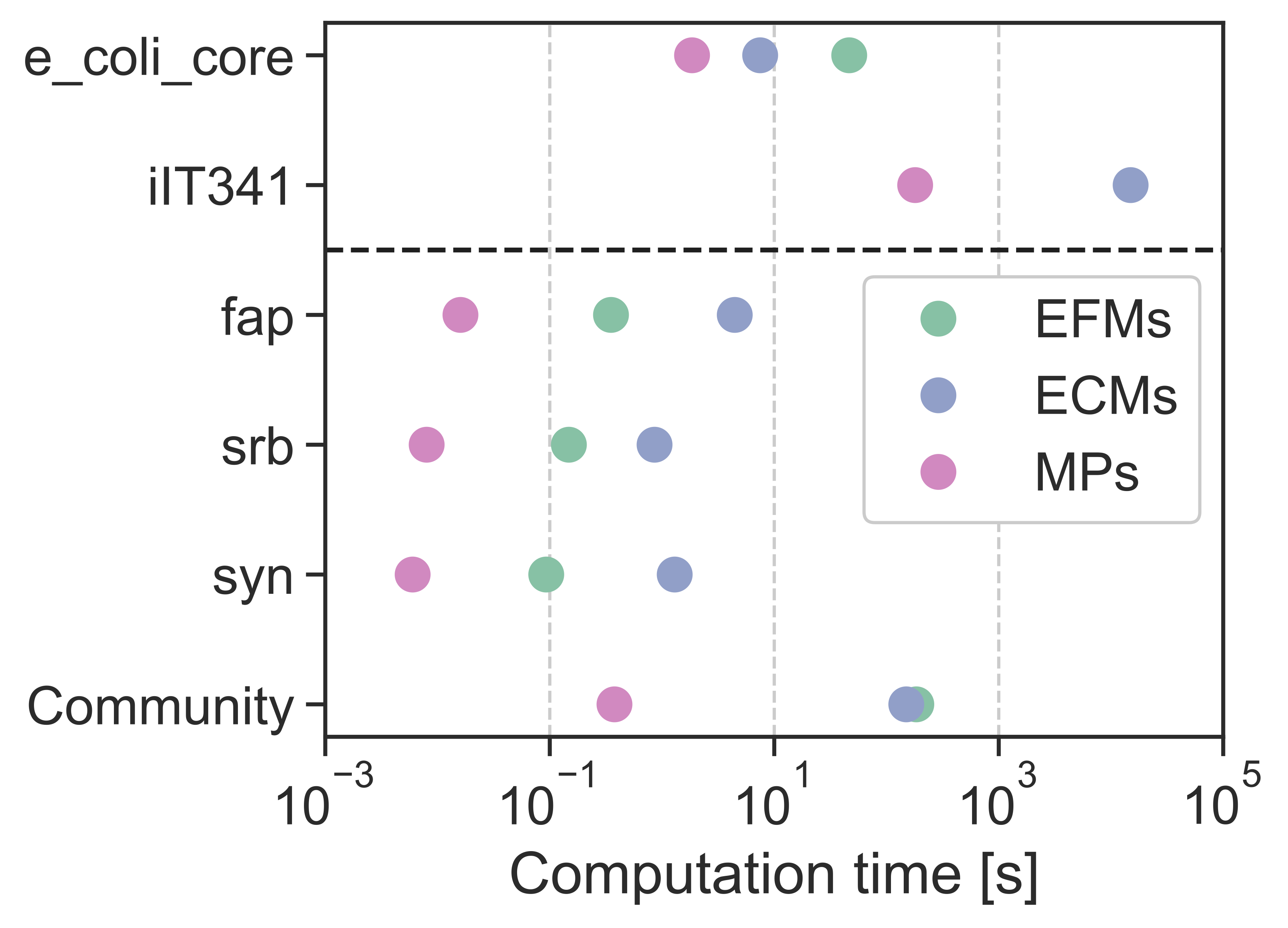

Supplement: S1 Fig — Computation time for enumerating all EFMs, ECMs, or MPs for all models analyzed in this study. Models used to analyze microbial species and models used to analyze a microbial community are separated by a dashed line. EFPs were extracted from EFMs or ECMs and therefore not enumerated separately. All enumerations except ECM enumeration for iIT341 were performed on the same laptop computer (see Methods for details). (TIFF) [file pcbi.1012472.s001.tiff]

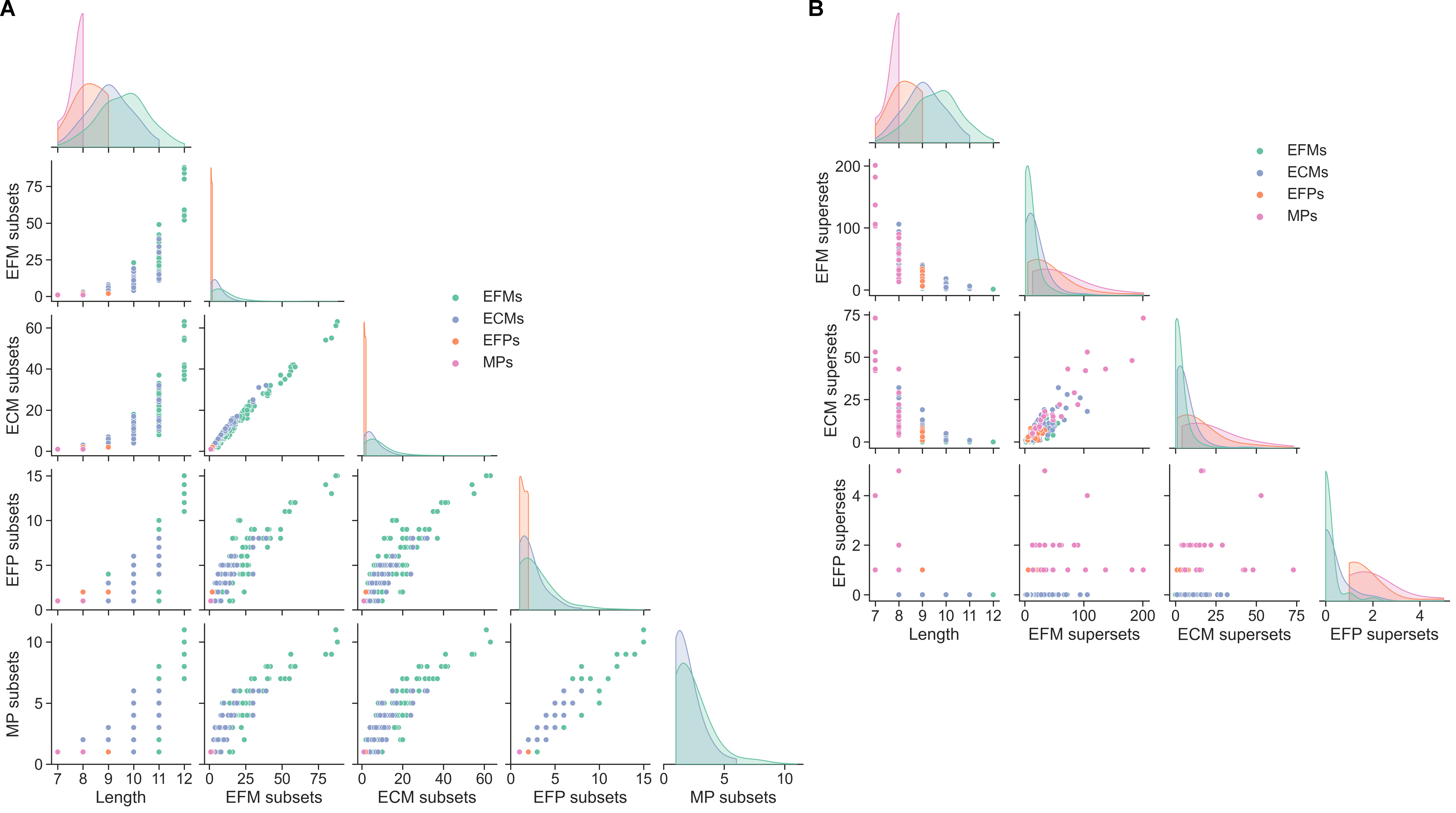

Supplement: S3 Fig — Pairwise relationships between (A) pathway length and number of EFM, ECM, EFP, and MP subsets and (B) pathway length and number of EFM, ECM, and EFP supersets for e_coli_core. (TIFF) [file pcbi.1012472.s003.tiff]

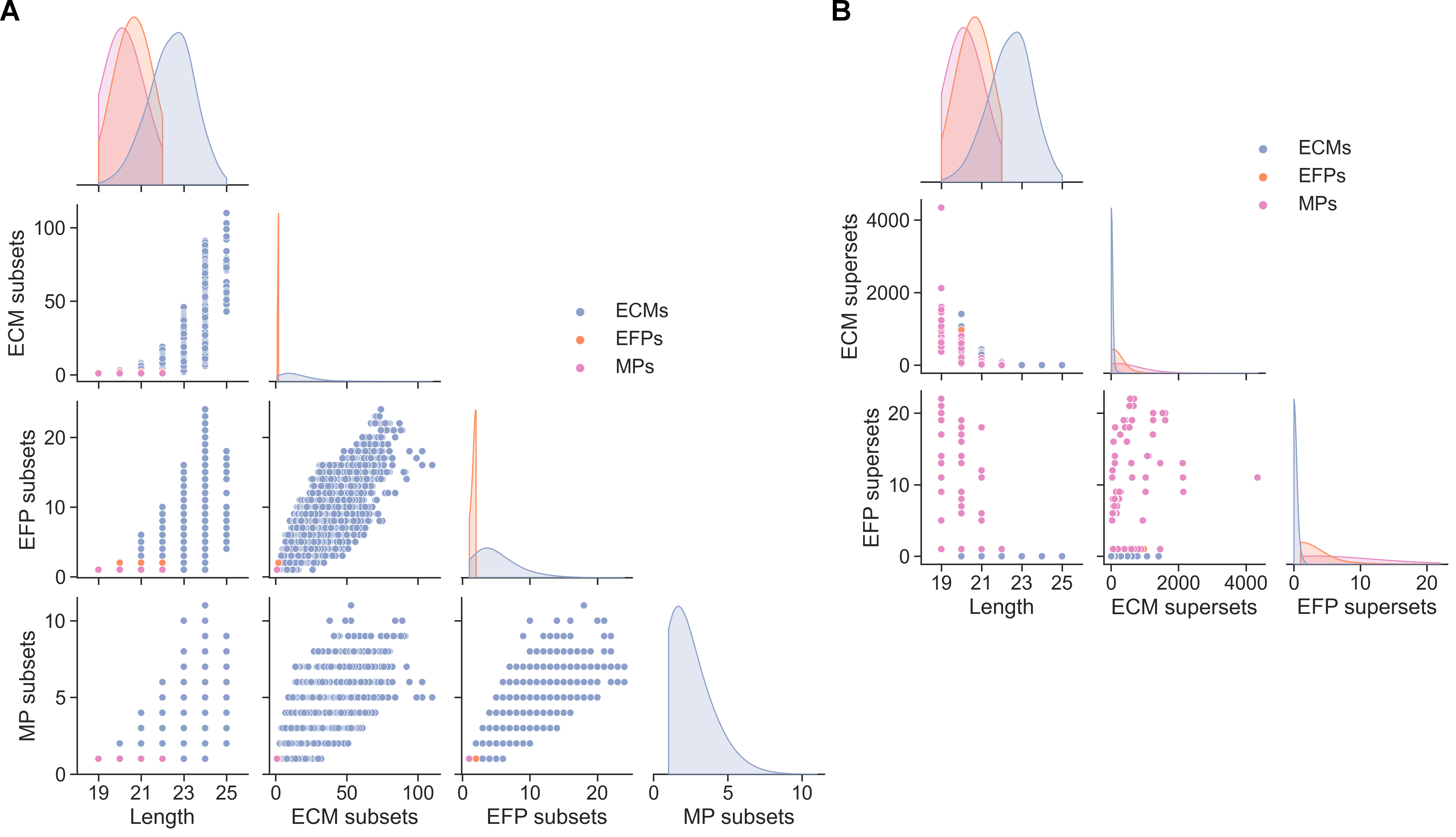

Supplement: S4 Fig — Pairwise relationships between (A) pathway length and number of ECM, EFP, and MP subsets and (B) pathway length and number of ECM and EFP supersets for iIT341. (TIFF) [file pcbi.1012472.s004.tiff]

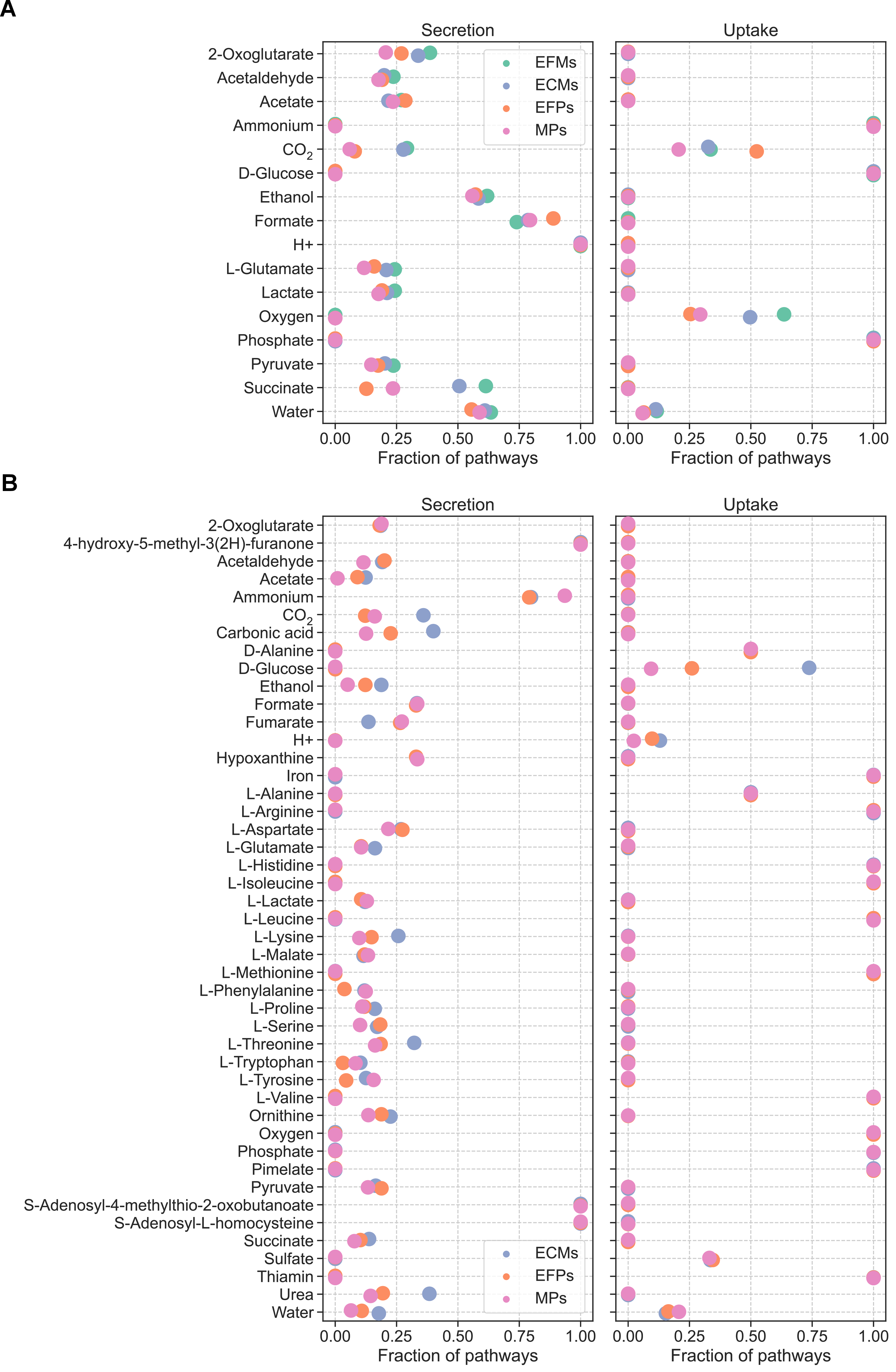

Supplement: S5 Fig — Metabolite exchange frequencies (fraction of pathways including secretion or uptake of each metabolite) for (A) e_coli_core and (B) iIT341. (TIFF) [file pcbi.1012472.s005.tiff]

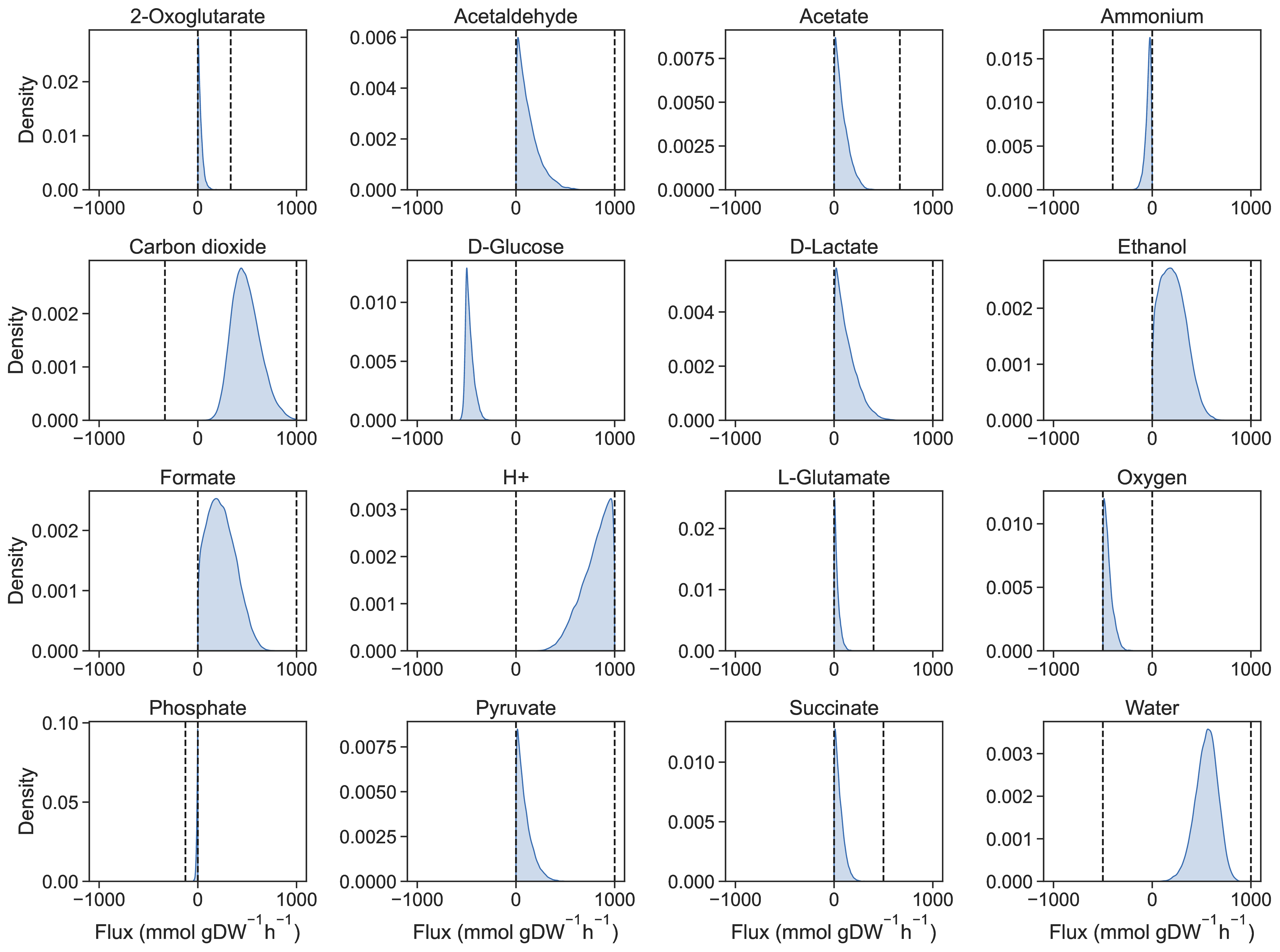

Supplement: S7 Fig — Flux probability distributions for metabolite exchanges in e_coli_core from 100,000 random flux vectors sampled with PTA. Dashed lines indicate feasible flux ranges from FVA. (TIFF) [file pcbi.1012472.s007.tiff]

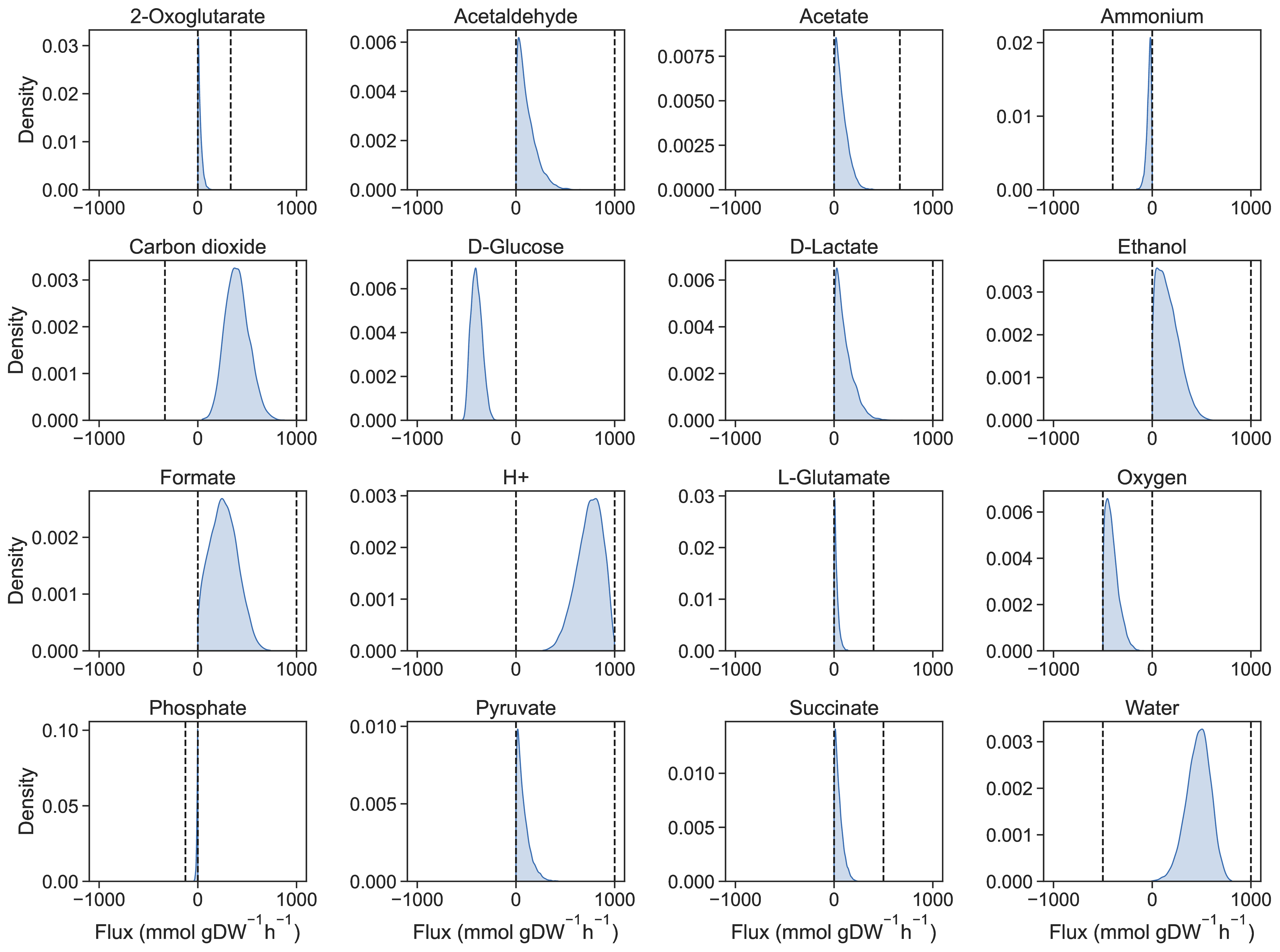

Supplement: S8 Fig — Flux probability distributions for metabolite exchanges in e_coli_core from 100,000 random flux vectors sampled with OptGP. Dashed lines indicate feasible flux ranges from FVA. (TIFF) [file pcbi.1012472.s008.tiff]

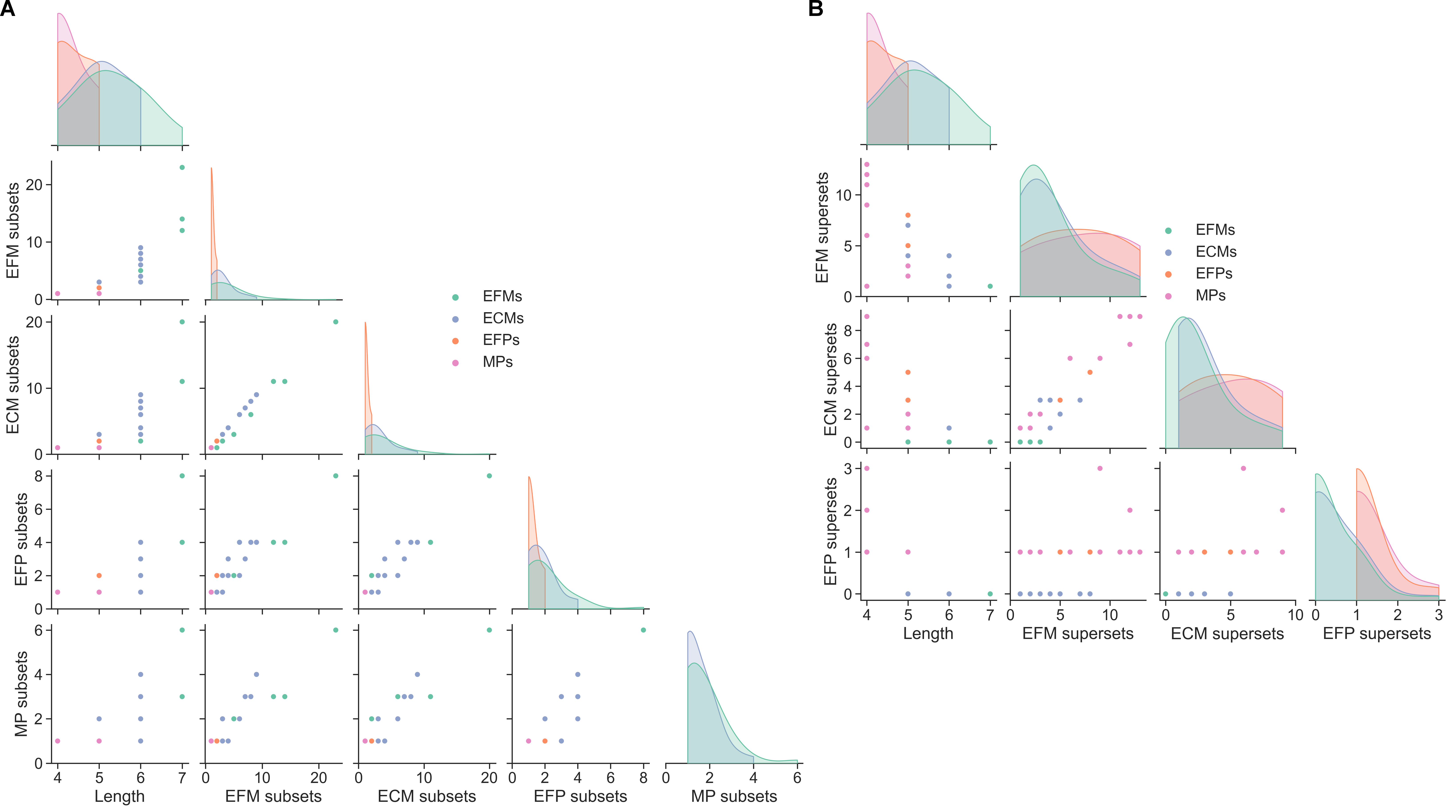

Supplement: S13 Fig — Pairwise relationships between (A) pathway length and number of EFM, ECM, EFP, and MP subsets and (B) pathway length and number of EFM, ECM, and EFP supersets for fap, srb, and syn individually. (TIFF) [file pcbi.1012472.s013.tiff]

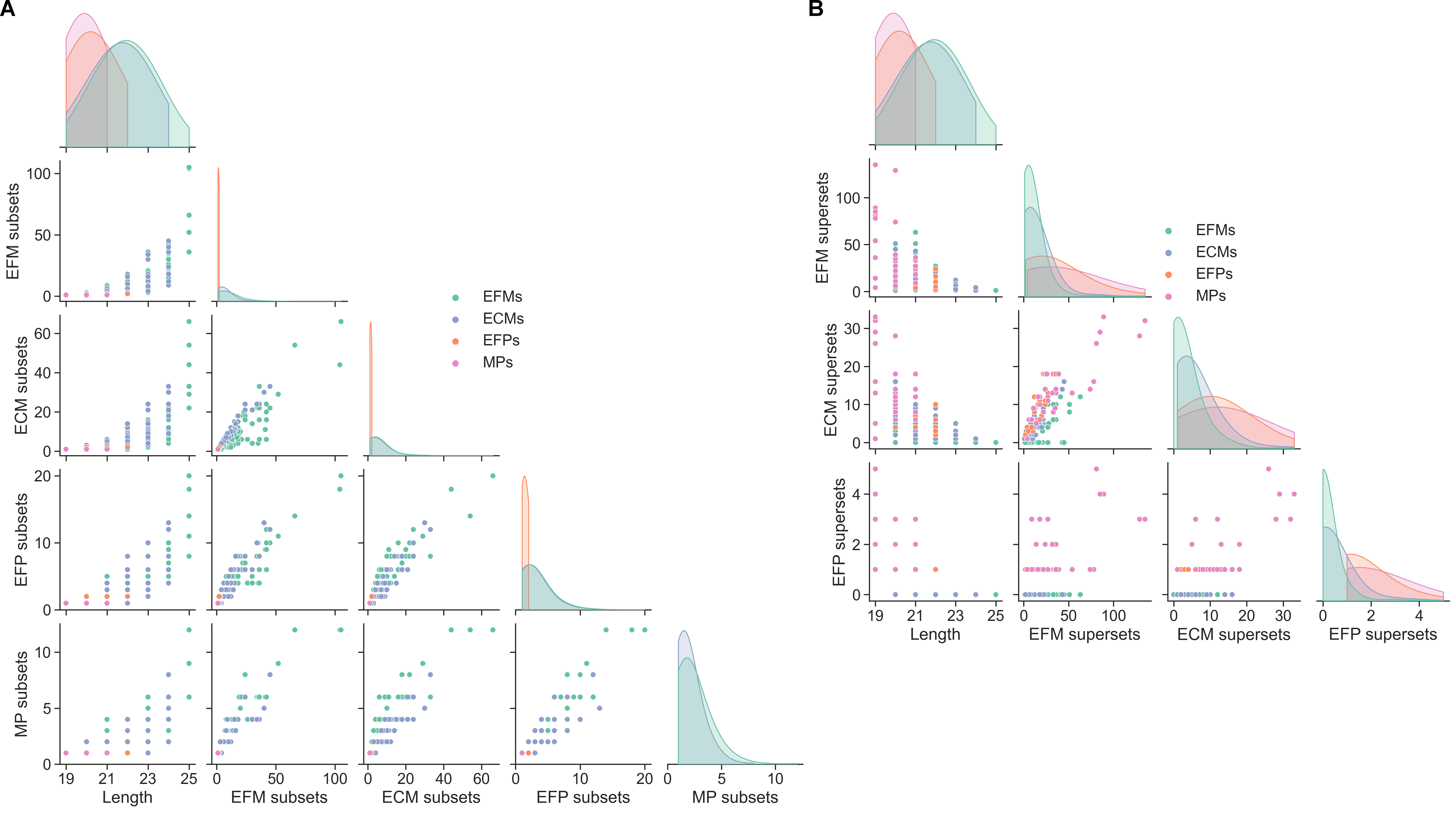

Supplement: S14 Fig — Pairwise relationships between (A) pathway length and number of EFM, ECM, EFP, and MP subsets and (B) pathway length and number of EFM, ECM, EFP, and MP supersets for the microbial community model. (TIFF) [file pcbi.1012472.s014.tiff]

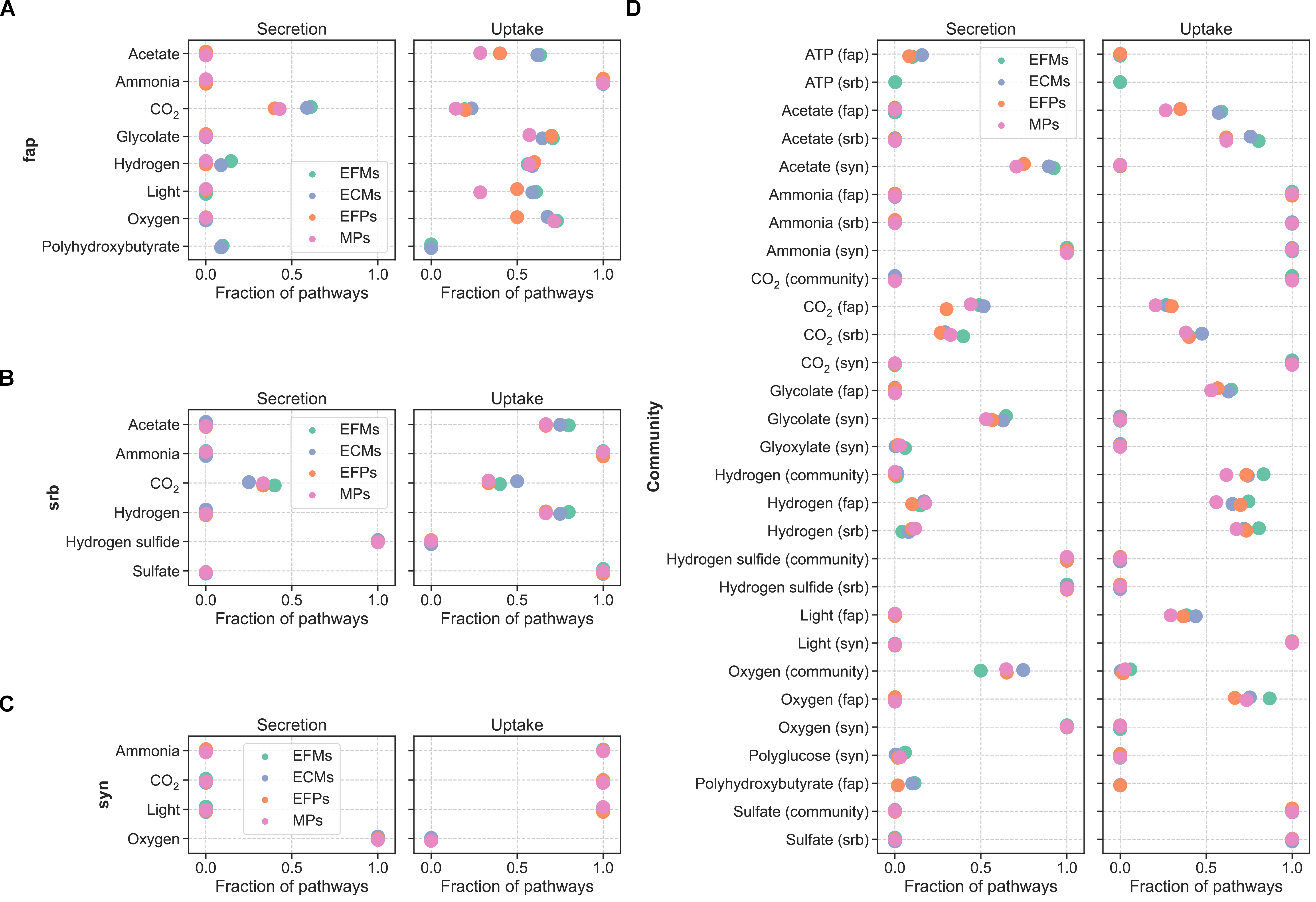

Supplement: S15 Fig — Metabolite exchange frequencies (fraction of pathways including secretion or uptake of each metabolite) for (A) fap, (B) srb, (C) syn, and (D) the microbial community model. (TIFF) [file pcbi.1012472.s015.tiff]

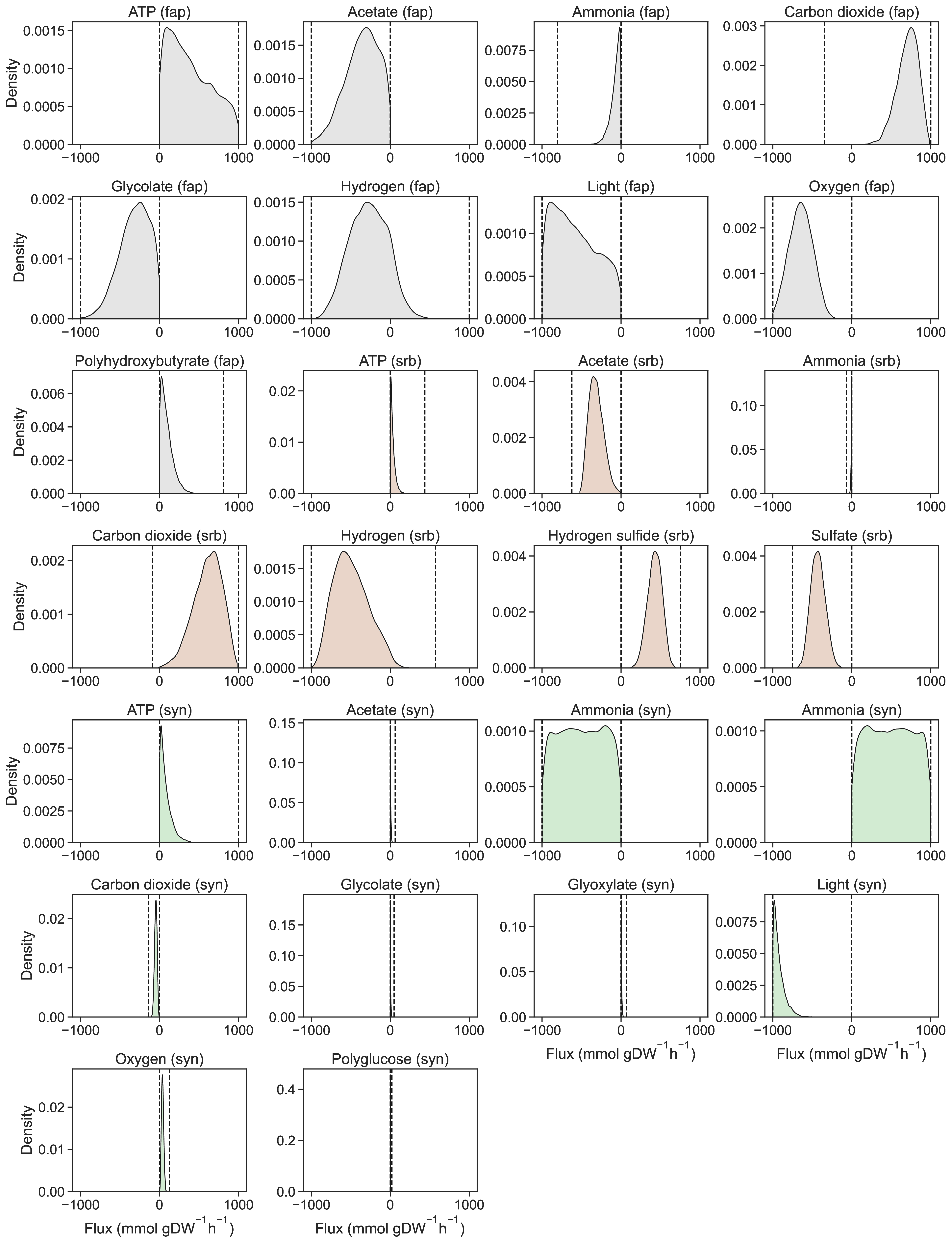

Supplement: S17 Fig — Flux probability distributions for metabolite exchanges in fap, srb, and syn individually from 100,000 random flux vectors sampled with OptGP. Dashed lines indicate feasible flux ranges from FVA. (TIFF) [file pcbi.1012472.s017.tiff]

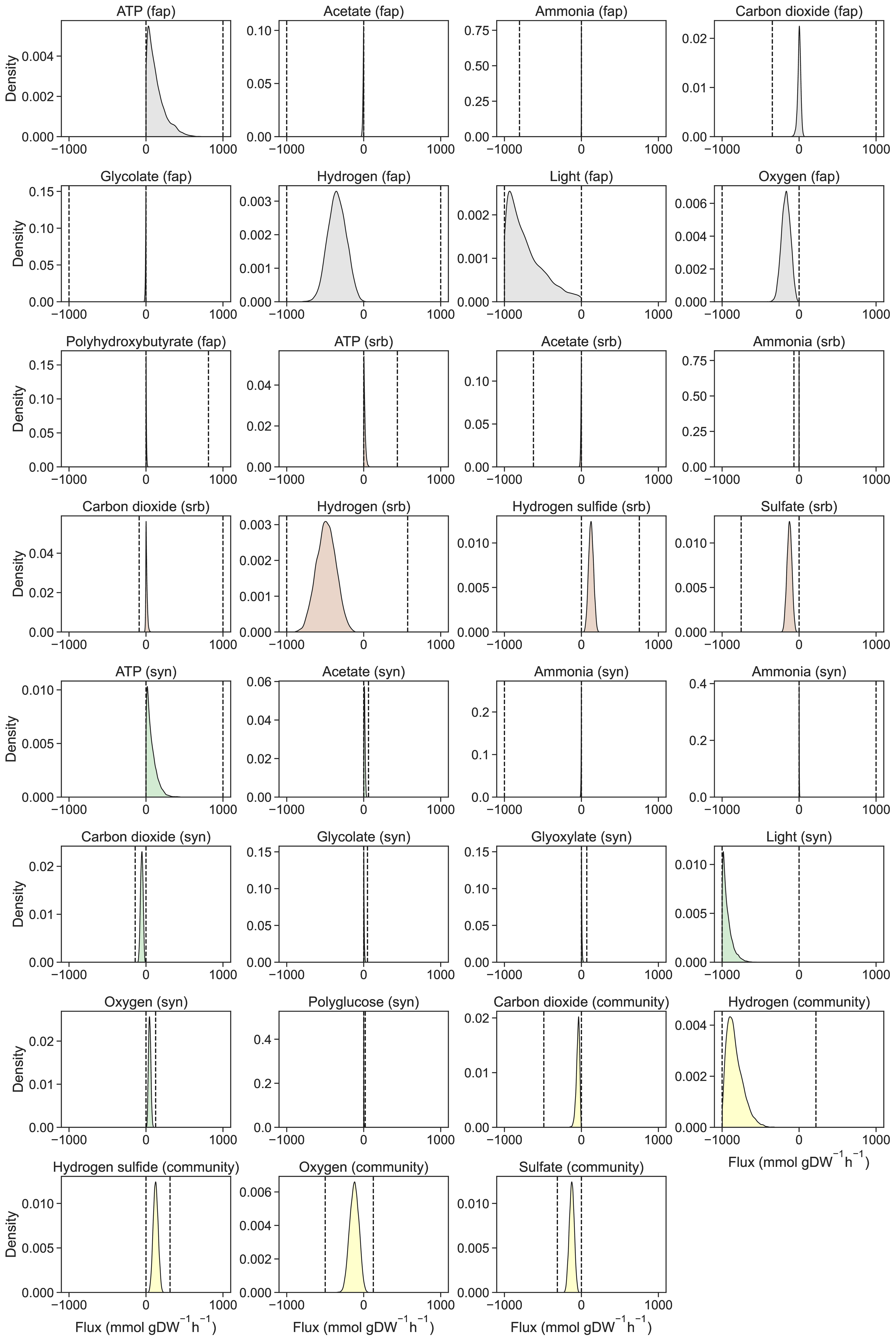

Supplement: S18 Fig — Flux probability distributions for metabolite exchanges in the microbial community model from 100,000 random flux vectors sampled with OptGP. Dashed lines indicate feasible flux ranges from FVA. (TIFF) [file pcbi.1012472.s018.tiff]
