# Supplementary material for: A hierarchy of metabolite exchanges in metabolic models of microbial species and communities
Source: PLoS Comput Biol. 2024 Sep 26;20(9):e1012472. doi: 10.1371/journal.pcbi.1012472 (PMC11460683; doi:10.1371/journal.pcbi.1012472)
Supplement: Attachment — Submitted filename: rebuttal_letter_plos.pdf [file pcbi.1012472.s019.pdf]

We thank the editor and the reviewers for their careful evaluation of our manuscript and for their constructive and positive comments. Taking all issues raised by the reviewers into account, we have revised the text of our manuscript and believe that it has been substantially improved. In particular, we have attempted to improve the readability of the manuscript by clarifying explanations and avoiding extensive use of abbreviations. Our point-by-point response to the reviewers' comments are included below (highlighted in red).

Reviewer #1

This study analyzes hierarchical relationships among four types of pathways for genome-scale metabolic models (GEMs) from the perspective of metabolic exchanges. The pathways examined include elementary flux modes (EFMs), elementary flax patterns (EFPs), elementary conversion modes (ECMs), and minimal pathways (MPs). The study demonstrated the general hierarchy as $P_{MP} \subseteq P_{EFP} \subseteq P_{ECM} \subseteq P_{EFM}$. They conducted computational experiments using small GEMs, e_coli_core and iIT341 as well as microbial community model based on e_coli_core. These experiments supported the efficiency of focusing on metabolic exchanges.

Thank you for thoroughly reviewing and summarizing our work.

The idea of focusing on metabolic exchanges and improving the scalability of pathway analysis is reasonable and efficient. However, the derived hierarchy seems obvious from the definitions.

While the hierarchical relationship between pathways may seem obvious in hindsight, we cannot find any reference to it in published work. Thus, despite extensive literature review, we were completely unaware of any such relationship when we first started using pathway enumeration to analyze metabolite exchanges. In fact, it was precisely this lack of information on the relationships between pathway methods, particularly in the emerging application to microbial metabolite exchanges, that prompted this study in the first place. The fact that the flux patterns of EFPs must be a subset of the flux patterns of ProCEMs (projected cone elementary modes), and that ProCEMs are equivalent to ECMs for the subnetwork of metabolite exchanges, has been demonstrated by Marashi et al. [1]. However, we would argue that the relationships between pathway definitions were otherwise largely unclear prior to our study.

We do completely agree with the reviewer that the hierarchical relationship can be deduced from the pathway definitions, but connecting existing definitions and proofs to arrive at this required substantial effort on our part. Again, we want to underscore that our role was to connect proofs made by others, in particular by Müller and Regensburger [2]. However, even if the pathway hierarchy was already obvious to some in the field, it had never been mentioned in any publication as far as we can tell. Also, as we argue in our manuscript, knowing how different pathway definitions relate to each other can be important both for computational efficiency and biological interpretation: it would have been valuable for us to know about this prior to our study.

Additionally, we demonstrate the pathway hierarchy computationally for the first time by systematically enumerating key pathway definitions in the same subnetworks and with identical constraints. It is important to note that the hierarchy holds as long as pathways are enumerated under exactly the same conditions, i.e., with the same network, subnetwork, and constraints.

[1] Marashi, S. A., David, L., & Bockmayr, A. (2012). Analysis of metabolic subnetworks by flux cone projection. Algorithms for Molecular Biology, 7, 1-9.
[2] Müller, S., & Regensburger, G. (2016). Elementary vectors and conformal sums in polyhedral geometry and their relevance for metabolic pathway analysis. Frontiers in Genetics, 7, 179906.

Below are points that seem to require additional explanation.

1.While the idea of ECMs and some other types of pathways is to ignore the internal reactions, lengths are defined for them. The definitions of the length for them should be clarified.

We have now clarified what we mean by pathway length in the text. Specifically, we explicitly state that we define the length of a pathway as the number of reactions participating in the pathway (i.e., reactions with non-zero flux). When we first introduce the pathway length on line 119, we now write "Comparing pathway lengths, i.e., the number of reactions participating in each unique growth-supporting pattern, we found that the number of shortest pathways was always the same for all pathway definitions." Also, in every subsequent use of the word length in this context, we now write "pathway length".

When analyzing subnetworks such as metabolite exchanges, in which reactions are not connected to each other, a pathway may also consist of reactions that are not directly connected (Fig. 1). However, we still refer to the number of reactions in such a pathway as its length. Possible alternative terms include "pattern" instead of "pathway" and "size" or "support" instead of "length". We prefer to keep using "pathway" and "length", as these are established and commonly used terms both in biology and in the field of formal metabolic pathway analysis. By keeping the concepts closer to biology, we also hope to make them easier to understand.

2.Metabolite exchange frequency should be formally defined.

We agree that metabolite exchange frequency is a crucial concept in our study that needs to be clearly defined. We have therefore edited the text to make it clear that we define the exchange frequency of a metabolite as the fraction of enumerated pathways in which the metabolite is exchanged. Specifically, we now define the exchange frequency several times throughout the manuscript, in the abstract and author summary, the main text, and the figure legends.

In the abstract, we now write "Metabolite exchange frequencies, defined as the fraction of pathways in which each metabolite was exchanged, were similar across pathway definitions, with a few specific exchanges explaining large differences in pathway counts".

In the author summary, we avoid the term “metabolite exchange frequency” altogether and write “Moreover, the fraction of pathways in which each metabolite was exchanged turned out to be remarkably consistent across pathway definitions despite large differences in pathway counts.”

From line 90 we now write “Moreover, metabolite exchange frequencies, i.e., the fraction of enumerated pathways that included exchange of each metabolite, were mostly stable across pathway definitions, showing that the same biological conclusions can be drawn from different methods.” We have also included this definition in the legends of Figs. 4 and 7.

3.Information regarding computation time appears to be lacking. Is it possible to enumerate the pathways focusing on metabolic exchanges for larger GEMs?

This is a good point and we have now included pathway enumeration running times in our results. The computation times for all models are shown in the new S1 Fig. and we list them in the text at first mention of each enumeration. For example, on line 107, we now write “The computation time for enumeration was 47 s for EFMs, 8 s for ECMs, and 2 s for MPs.”

The computation times are in line with our discussion of the relative scalability of enumeration methods, and they highlight the importance of understanding how pathways relate to each other to make enumeration more scalable. For example, EFM enumeration was as fast as or even faster than ECM enumeration for the models used to analyze the microbial community, despite many more EFMs than ECMs for the community model. It would be great if some or all of the advantages of the very efficient EFM enumeration methods could be used for other pathways as well.

We do show that ECM and MP enumeration is feasible in a genome-scale model, albeit one that is small compared to most GEMs published today (iIT341). Pathway enumeration of course scales very poorly in general, but we make the argument that MPs in particular can be enumerated in larger models through a combination of minimizing the number of pathways, efficient enumeration and sampling algorithms, and a flexible subnetwork definition. This has already been shown in a previous study, in which we demonstrated scaling of MP analysis to genome-scale networks and subnetworks with thousands or tens of thousands of reactions [3]. Here, we argue that clarifying the relationship between pathway definitions can help interpret enumerated pathways and potentially improve scalability for other definitions than MPs as well.

[3] Øyås, O., & Stelling, J. (2020). Scalable metabolic pathway analysis. bioRxiv, 2020-07.

Reviewer #2

This study investigated and compared different pathway analysis methods (elementary flux modes, elementary conversion modes, elementary flux patterns, and minimal pathways), using metabolic models of microbial species from small size to genome-scale, as well as community level model of a microbial consortium. The authors mainly analyzed metabolite exchanges between species, or between species and environment, and found that different pathway

definitions for metabolic networks or subnetworks were related through a hierarchy. Thank you for doing this work and adding to our knowledge base in this field. Here are some comments for further refinement of the manuscript.

Thanks a lot for reviewing and recognizing the contribution of our manuscript to the field.

1.) The authors should introduce in more detail about reconstruction process of the microbial community model, e.g. what modeling method was used, how to construct biomass reaction…

We agree that our description of the microbial community model construction was a bit too minimal. We have tried to clarify the text and now go into more detail on how the models of individual microbes were reconstructed from the supplementary information of Taffs et al. [4] and how the individual models were merged into a microbial community model. The description of the model reconstruction from line 405 now reads as follows:

"Using COBRApy, we built individual models of fap, srb, and syn by adding the reactions listed for the compartmentalized daylight scenario in the supplementary information of Taffs et al. to the models. We added exchange reactions for all metabolites listed as external and manually identified sink and demand reactions as defined by Thiele et al. The community model was made by merging the individual models into a new model and connecting them through exchanged metabolites in a shared compartment. Specifically, the exchange reactions of the individual models were connected to the shared metabolites and new exchange reactions between the shared compartment and the environment were created, allowing the intermicrobial and environmental exchanges described in Fig. 1A of Taffs et al. A pseudo-metabolite consisting of equal shares of biomass from each of the individual microbes was constructed and used as the substrate in a community biomass reaction, thus requiring balanced growth of all three microbes."

[4] Taffs, R., Aston, J. E., Brileya, K., Jay, Z., Klatt, C. G., McGlynn, S., ... & Carlson, R. P. (2009). In silico approaches to study mass and energy flows in microbial consortia: a syntrophic case study. *BMC systems biology*, *3*, 1-16.

2.) For srb model, does it have 209 EFMs (line 140)?

Thank you for pointing this out. You are of course correct that srb does not have 209 EFMs and this has now been corrected in the text (we actually ended up removing this specific listing of pathways) . Our apologies for the mistake.

Reviewer #3

Overall comment:

The manuscript is highly technical, but an important study in the field. The hierarchical relationship between pathway definitions is well-explained and supported by extensive

computational analysis. I confess I had to read several sections multiple times and had a “cheat sheet” with the definitions of EFMs, ECMs, EFPs on the side since each abbreviation is mentioned close to 100 times each. I understand this is the result of the highly technical nature of this manuscript and while it’s not a dealbreaker for me, any improvements to the readability of the manuscript would be welcome. In particular, consider adding more descriptive captions to the figures across the manuscript, In particular figures 3 and 4 can use caption improvements the most. The amount of data provided in supplementary materials is commendable and allows for a deep dive into how these studies were performed. I also appreciate seeing the clear statement of the author's contributions with a lot of detail. I recommend that this manuscript be accepted for publication with only minor comments below.

We are grateful to the reviewer for the detailed review and appreciation of our work.

Also, thanks for the helpful suggestions, which we think have improved the manuscript. As stated earlier, we have now made an effort to make the text more readable, especially by avoiding listings of pathway abbreviations to the extent possible.

Furthermore, we have updated all the figure captions to make them more clear. To be specific, we have shortened, rewritten, and reorganized all the captions to avoid repetition and to make them easier to read and understand (perhaps most notably for Figs. 3 and 4).

Detailed comments:

Abstract:

The abstract provides a concise synopsis of the study question, methodology, findings, and conclusion is given in the abstract. The main conclusions are emphasized along with their importance. However, can the authors add a brief explanation of the study's more general implications?

Absolutely! We have now modified abstract, author summary, introduction, and discussion to focus more on the implications of this study for metabolic pathway analysis, and its potential for applications to cell factories and to help understand biology.

At the end of the abstract, we now write “Our results also signal wider biological implications, facilitating detailed and interpretable analysis of metabolite exchange and other subnetworks in fields such as metabolic engineering and synthetic biology”, and at the end of the author summary we write “In summary, our work shows how pathway definitions and their metabolite exchange predictions are related to each other, and it facilitates scalable and interpretable pathway analysis with applications in fields such as metabolic engineering.”

We expand on these general statements in two new paragraphs, one towards the end of the introduction (line 62) and one at the start of the discussion (line 379).

Introduction:

Lines 4-5: "The growing availability of genomes and other omics data has enabled metabolic network reconstruction in silico, giving rise to genome-scale metabolic models (GEMs) that are usually formulated as constraint-based models (CBMs) to allow scaling." A reference to a review paper on the history and development of GEMs and CBMs would be beneficial here.

We agree that this is important and did already include a reference to Bordbar et al. [5] at the end of this sentence. This review paper includes a summary of the history of GEMs and CBMs, but it is ten years old and we have therefore added a more recent reference to Fang et al. [6].

[5] Bordbar, A., Monk, J. M., King, Z. A., & Palsson, B. O. (2014). Constraint-based models predict metabolic and associated cellular functions. *Nature Reviews Genetics*, *15*(2), 107-120.
[6] Fang, X., Lloyd, C. J., & Palsson, B. O. (2020). Reconstructing organisms in silico: genome-scale models and their emerging applications. Nature Reviews Microbiology, 18(12), 731-743.

In Figure 1, make sure that all abbreviations are defined in the caption.

Thank you, we have now modified the caption of Fig. 1 to define all abbreviations as follows: "The elementary flux modes (ECMs), elementary flux patterns (EFPs), and minimal pathways (MPs) of the network."

Overall, the manuscript introduction does a good job of explaining pathway definitions and their hierarchical relationships clearly. However, can the authors provide more background on the practical implications of these findings for metabolic engineering and synthetic biology?

Again, thank you for your constructive suggestions. As summarized in our previous answer above, we now include a more in-depth introduction and discussion of the potential implications of scalable pathway analysis for metabolic engineering and synthetic biology in particular, specifically through new paragraphs in the introduction and the discussion.

Results and Discussion:

Lines 113-116: "For each exchanged metabolite, we computed the frequency of uptake and secretion in EFMs, ECMs, EFPs, and MPs as well as differences in frequency between pathway definitions." Can teh authors provide further detail on how the frequency computation is performed?

This point was also raised by reviewer #1 (see our answer above) and we now realize that we did not clearly state what we meant by "metabolite exchange frequency". Throughout the manuscript, we have clarified that exchange frequency is defined as the fraction of enumerated pathways in which a metabolite is exchanged, and we have explained the computation in more detail in methods.

Line 332, correct the typo in "lexicograhic" to "lexicographic."

Thanks, we have corrected this.

According to the study, every metabolic network or subnetwork can be generalized to have a hierarchical link between pathway descriptions. What are the potential exceptions and/or limitations? Any alternative interpretations?

Thank you for your thoughtful questions. These are interesting topics for further research and we have expanded on the possible implications and limitations of the pathway hierarchy. As noted above in our response to reviewer #1, but worth stating again, is the importance of identical conditions when enumerating pathways. That is, the pathway hierarchy holds as long as pathway definitions are enumerated for equivalent flux spaces, i.e., using equivalent networks, subnetworks, and constraints. We have also modified the discussion to underscore this, writing that the hierarchy "holds as long as pathways are enumerated under exactly the same conditions, i.e., with equivalent networks, subnetworks, and constraints" on line 283.

Can you discuss how the hierarchical relationship between pathway definitions can/will impact future research in the field of metabolic network analysis?

Thanks again. We now elaborate on the possible impacts of our findings on future research in more detail at the end of the discussion. In summary, we think that the main impact of our findings could be to help interpret pathway definitions in relation to each other as well as in terms of biology, and we hope this can help make pathway analysis more accessible and scalable. We also want to start a conversation about unification of existing frameworks for pathway analysis, hopefully to make methods more scalable and accessible as well as to aid researchers in choosing the best methods for their application.

The results of the computation studies are compelling, but can the authors empathize with the biological significance of some conclusions? For example, what new insights (if any) do these studies provide into how microbial communities in industrial settings or natural environments operate?

We agree that the whole point of enumerating pathways should be to understand biology, and we now expand on possible implications for biology in industrial and natural settings in both introduction and discussion. While it is challenging to draw general conclusions on metabolite exchanges in microbial communities and their importance for biology and ecology based on our results, we do interpret specific metabolite exchanges identified in this study in terms of biology:

From line 321, we argue that overrepresentation of glucose uptake and underrepresentation of ammonium secretion in ECMs relative to MPs can be explained by the minimality requirement of MPs. Also, from line 349, we argue that CO_2 uptake observed in pathways but not in samples for *E. coli* reflects real biology: *E. coli* does indeed encode the enzymes needed to take up and

utilize CO_2 from the environment, but this metabolic mode of operation likely corresponds to a very small fraction of the feasible flux space.

The manuscript addresses the scalability of various enumeration methods and even mentions that in some cases you could not compute the full set for a given model, but a more thorough description of their computing effectiveness and usefulness for large-scale models would be beneficial.

Thank you for making this important point, which was also raised by reviewer #1. We have now included computation times for pathway enumeration in our results. The computation times for all models are now listed in the text at first mention and shown in the new S1 Fig.

We do show that ECM and MP enumeration is feasible in a relatively small genome-scale model (iIT341). However, we were not able to enumerate ECMs for any genome-scale models larger than iIT341. We have previously shown that MP analyses scale to networks and subnetworks with tens of thousands of reactions [3], and we now argue that clarifying the relationship between pathway definitions can potentially improve interpretation and scalability for other pathways than MPs as well, e.g., by adapting efficient methods for EFM enumeration.

As an example of how difficult scaling of ECM enumeration can be with current methods, we have some results for the genome-scale *E. coli* model iJR904 (1075 reactions) that did not make it into the manuscript. Specifically, we attempted to reproduce enumeration of 12 ECMs in a tiny subnetwork of iJR904 consisting only of oxygen and glucose exchange [7]. Using the indirect method and distributing jobs across 10 nodes with 32 cores each, this was expected to take about 14 hours [6], but we had to shut it down after 8.5 days due to time restraints. For comparison, the 12 ECMs correspond to one MP that was found in less than one second.

[7] Clement, T. J., Baalhuis, E. B., Teusink, B., Bruggeman, F. J., Planqué, R., & de Groot, D. H. (2021). Unlocking elementary conversion modes: Ecmtool unveils all capabilities of metabolic networks. Patterns, 2(1).
